# Derivation of Induced Pluripotent Stem Cells from Fetal Human Skin Fibroblasts

**Published:** 2010-07

**Authors:** S.P. Medvedev, A.A. Malakhova, E.V. Grigor’eva, A.I. Shevchenko, E.V. Dementyeva, I.A. Sobolev, I.N. Lebedev, A.G. Shilov, I.F. Zhimulev, S.M. Zakian

**Affiliations:** Institute of Cytology and Genetics, Siberian Branch, Russian Academy of Sciences; Research Institute of Medical Genetics, Siberian Branch, Russian Academy of Medical Sciences; Institute of Chemical Biology and Fundamental Medicine, Siberian Branch, Russian Academy of Sciences; Research Center of Clinical and Experimental Medicine, Siberian Branch, Russian Academy of Medical Sciences

**Keywords:** induced pluripotent stem cells, reprogramming, retroviral vectors

## Abstract

The isolation and study of autologous human stem cells remain among the most urgent problems in cell biology and biomedicine to date. Induced pluripotent stem cells can be derived from human somatic cells by the overexpression of a number of genes. In this study we reprogrammed fetal human skin fibroblasts by transduction with retroviral vectors carrying murine *Oct4 * , * Sox2 * , * Klf4 * , and * c–Myc* cDNAs. As a result, cells with the protein expression and gene transcription pattern characteristic of human embryonic stem cells were derived. These induced pluripotent cells are capable of differentiation * in vitro * into the ectoderm, mesoderm, and endoderm derivatives.

## INTRODUCTION

Induced pluripotent stem cells are a unique model for studies in many fields of biomedicine, such as the molecular basis of human cell pluripotency and reprogramming (processes occurring in early embryogenesis) [1]. Broad prospects are opening up for the application of induced pluripotent stem cells in toxicology and pharmacology, as well as in regenerative medicine [2–4].

In this work we intended to prepare stable lines of pluripotent cells from human embryonic fibroblasts (HEFs) by their transduction with retroviral vectors carrying murine * Oct4, Sox2, Klf4, * and * c–Myc * genes.

**Fig. 1:**
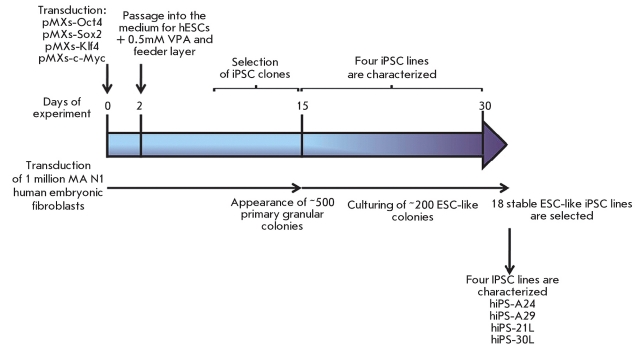
Schematic representation of an experiment on the preparation of induced pluripotent stem cells from human embryonic fibroblasts.

**Fig. 2:**
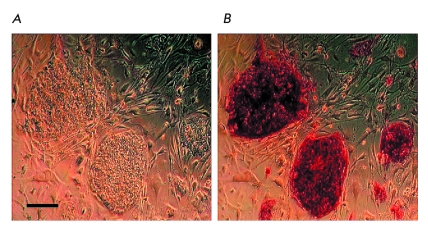
(A) morphology of iPSC colonies derived from hEFs; (B) staining of the iPSC colonies demonstrating the expression of alkaline phosphatase (one of the pluripotent cell markers). Scale 100 µm.

**Fig. 3:**
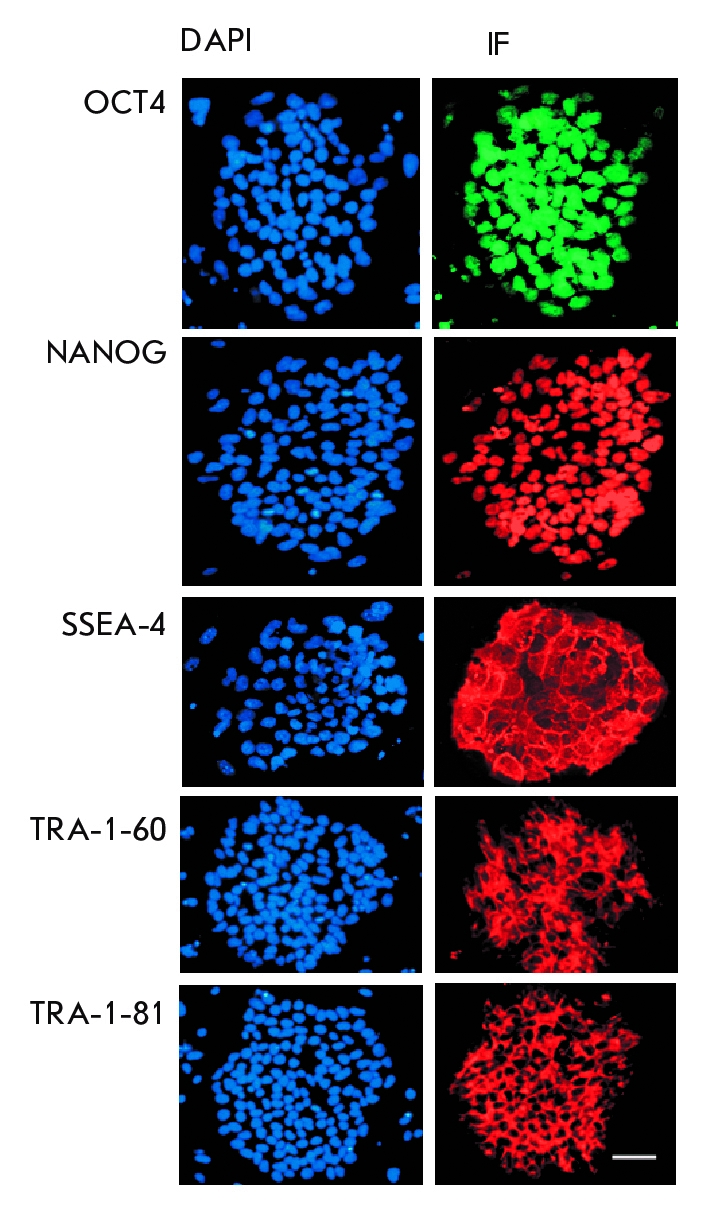
Immunocytochemical staining (IF) of iPSC colonies with antibodies against the transcription factors OCT4 (green) and NANOG (red) and against the surface antigens SSEA-4, TRA-1-60, and TRA-1-81 (red). Cell nuclei are counterstained with DAPI (blue). Scale 100 µm.

**Fig. 4:**
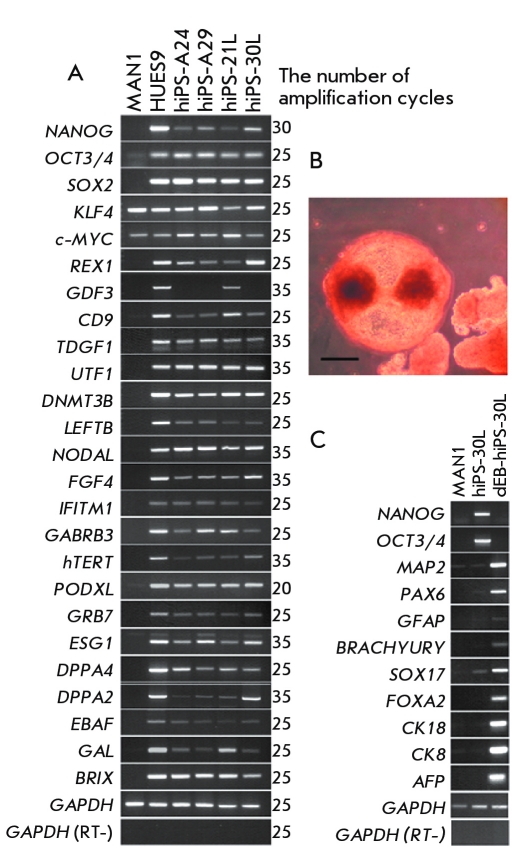
(A) RT-PCR assay of gene transcripts, which are the markers of human embryonic stem cells, in embryonic fibroblasts (MA N1), human embryonic stem cells (HUES9), and four iPSC lines: hiPS-A24, hiPS-A29, hiPS-21L, and hiPS-30L; (B) embryoid bodies formed following the passage of hiPS-30l iPSC cells into a liquid medium, scale 100 µm; (C) RT-PCR assay of gene transcripts, which are the markers of three germ layers (ectoderm, mesoderm, and endoderm), following the differentiation of the hiPS-30L clone via the embryoid body formation.

The MA N1 HEF line (ninth week of gestation) was used as the starting cell population. The fibroblasts were transduced with retroviruses prepared by the cotransfection of the constructs pMXs–Oct4, pMXs–Sox2, pMXs–Klf4, and pMXs–c–Myc carrying the murine * Oct4, Sox2, Klf4, and c–Myc * cDNAs [5] and a plasmid carrying the vesicular stomatitis virus (VSV) glycoprotein G (VSV–G) into the cells of the Phoenix HEK293 packaging line carrying the viral *Gag * and * Pol genes * . The retroviral vector carrying a pMX–GFP construct (Cell Biolabs, United States) encoding the green fluorescent protein (GFP) was used as the control. About 1 million fibroblasts were used in the experiment. Two days (48 h) after transduction, the cells were seeded onto the feeder (mitotically inactive murine fibroblasts treated with mitomycin C) grown in a medium for human ESC containing 0.5mM of 2–propylvaleric acid (valproic acid, VPA). At the second week after transduction, about 500 granular cell colonies appeared that differ in morphology from fibroblasts. However, these colonies were negative when stained for alkaline phosphatase (AP), one of the markers of pluripotent cells. It is likely that these cells corresponded to early reprogramming stages [6]. We continued selecting ESC–like clones by morphology for 15–30 days after transduction. The scheme of the experiment is shown in Fig. 1. Of the 200 clones subjected to the following cultivation, AP–positive ones began to appear. Totally, we obtained 18 stable ESC–like lines, four of which (hiPS–A24, hiPS–A29, hiPS–21L, and hiPS–30L) were characterized in detail. Cells of these lines have a high nucleus–cytoplasm ratio; form dense colonies, i.e., demonstrate morphological features of human ESC (Fig. 2A); and are AP–positive (Fig. 2B). Using PCR, we ensured that all four lines contained inserts of the used retroviral constructs (pMXs–Oct4, pMXs–Sox2, pMXs–Klf4, and pMXs–c–Myc). Using immunocytochemistry, we found that these cells expressed the following pluripotent cell markers: TRA–1–60, TRA–1–81, and SSEA–4 surface antigens, as well as NANOG and OCT4 transcription factors (Fig. 3). Using RT–PCR, we examined a transcription of 25 gene markers of human ESCs (Fig. 4A). All four clones were very similar in their expression pattern to the human ESC HUES9 line taken as a positive control. iPSCs derived from ESFs express the robust pluripotent cell markers, such as the genes * OCT4, NANOG, SOX2, FGF4, REX1, DNMT3B, NODAL, etc. * (Fig. 4A). The only difference was found in the transcription of the * GDF3 gene * . Of the four tested iPSC lines, this gene is only transcribed in hiPS–21L. Among the 25 gene markers, the transcription of *Klf4 * and * c– Myc * is only observed in MA N1 fibroblasts (Fig. 4A) . * The iPSCs* hiPS–A24, hiPS–A29, hiPS–21L, and hiPS–30L cells form embryoid bodies in suspension culture (F * ig. 4B * ). An RT–PCR assay of the cells formed during the growth of embryoid bodies showed the presence of markers characterizing the derivatives of all three germ layers: ectoderm ( * MAP2, PAX6, and GFAP * ), mesoderm ( * BRACHYURY* ), and endoderm ( * SOX17, FOXA2, CK8, CK18, and AFP * ) (F * ig. 4C* ). An immunocytochemical analysis of cells from disaggregated embryoid bodies revealed various cell derivatives expressing ectodermal ( β –III–tubulin and GFAP), mesodermal (collagen I and fibronectin), and endodermal ( α –fetoprotein and cytokeratin 18) markers (F * ig. 5 * ). Cells were also found that express both endodermal and mesodermal markers GATA6, as well as those expressing the protein nestin, which is a marker of both ectodermal and mesodermal derivatives (F * ig. 5 * ). Thus, we have shown that the obtained human iPSCs possess a broad differentiation potential * in vitro * . Karyotyping of the obtained iPSC lines showed a normal chromosomal set46, XY.

**Fig. 5:**
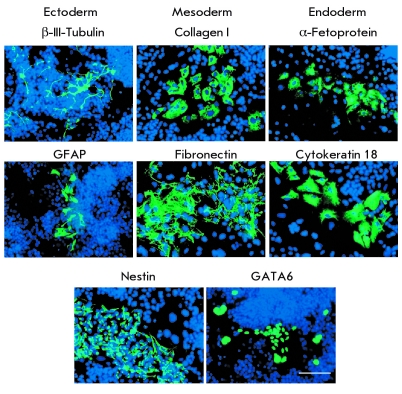
Expression of gene markers characterizing three germ layers (ectoderm, mesoderm, and endoderm) in spontaneous iPSC differentiation in embryoid bodies derived from hEFs. Scale 100 µm.

Thus, we have shown that the expression of the murine * Oct4, Sox2, c–Myc* , and * Klf4 * genes in human cells can result in their reprogramming with the formation of stable iPSC clones that are very similar in their features to human ESCs. Reprogramming fibroblasts using retroviral constructs is a replicable method allowing the accumulation of a great amount of pluripotent cell clones in a relatively short time.
